# Interactive effects of lysine restriction, vitamin fortification, and insoluble fiber on intestinal morphometry in broilers fed reduced-crude-protein diets

**DOI:** 10.1016/j.vas.2026.100574

**Published:** 2026-01-21

**Authors:** Ahmad Salahi, M.H. Shahir, Iraj Jafari Anarkooli, Zahra Abdi

**Affiliations:** a Department of Animal Science, Faculty of Agriculture, University of Zanjan, Iran; b Department of Animal Science, Agriculture Faculty, University of Zanjan, Iran; c Department of Anatomical Sciences, Faculty of Medicine, Zanjan University of Medical Sciences, Zanjan, Iran

**Keywords:** Intestinal morphology, Crude CP reduction, Lysine restriction, Vitamin and fiber interaction, Jejunal Histomorphometry

## Abstract

This 2 × 2 × 2 + 1 factorial trial examined digestible lysine:CP (6.2 vs. 5.7%), vitamins (standard vs. enhanced), and fiber (0 vs. 0.5% Arbocel®) effects on jejunal morphometry in 756 male Ross 308 broilers (d10–40) fed 12% reduced-CP diets vs. control. Protein and lysine reduction had limited effects on overall intestinal dimensions, though jejunal length declined (*P* = 0.041). Insoluble fiber significantly enlarged cecal surface area (*P* = 0.023, +15.2%). Vitamin × fiber and lysine × fiber interactions significantly influenced intestinal segment weights and lengths (*P* < 0.05). CP reduction markedly decreased epithelial length (-12.3%), villus height (-10.5%), villus surface area (VSA) (-18.7%), and wall thickness (*P* ≤ 0.001), while lysine restriction alone had minimal impact except on crypt number (*P* = 0.002). Vitamin fortification enhanced epithelial length (+8.4%), villus height (+9.1%), and enterocyte count (+11.2%), in HV2F0 birds. Synergistic fiber × vitamin and fiber × lysine interactions significantly modulated epithelial structure (*P* < 0.05). AI-based ImageJ analysis outperformed Cell^ A, detecting 5.2% shorter villi and 10.9% deeper crypts, confirming its superiority in microstructural assessment. CP and lysine reductions altered goblet cell number and crypt dimensions, whereas fiber increased goblet cell area and ratios (+14.6%). Vitamin effects on goblet metrics were modest but significant (*P* = 0.032). In conclusion, while CP and lysine reduction perturbed jejunal microstructure, synergistic supplementation with vitamins and insoluble fiber effectively supported mucosal integrity, highlighting their compensatory role in maintaining gut health under protein-reduced conditions.

## Introduction

The avian gastrointestinal tract exhibits remarkable phenotypic plasticity, adapting morphology and function to dietary composition, energy demands, and environmental cues. This adaptability, well-documented across vertebrates and optimizes nutrient absorption efficiency under varying dietary conditions, as seen in seasonal gut length variations and fiber-induced cecal changes in both wild and domestic birds (Karasov, 1996; McWilliams & Karasov, 2014). Broiler chickens, with their rapid growth and high metabolic rates, offer a controlled model for studying these intestinal adaptations and their implications for efficient production.

Nutritionists are not merely feeding animals—they play a critical role in shaping national and global food systems. This pathway starts from the chickens' intestines and ultimately impacts human intestines and health. The gut, recognized as the largest immune organ and a key site of microbial colonization, is considered one of the body's three brains: the cephalic (86 billion neurons), cardiac (40,000 neurons), (Sousa et al., 2017) and enteric nervous system (ENS), with over 100 million neurons in the small intestine (Rosenberg et al., 2021). In poultry, the gut and liver represent 4–5 % of body weight, while the GI tract accounts for ∼20 % of dietary energy use, mainly for enterocyte protein turnover and ion transport (Kelly & McBride, 1990). Glutamate and glutamine are major enterocyte fuels (He et al., 2019), and much of the absorbed amino acids are utilized by the intestinal mucosa through anabolic and catabolic routes rather than entering the portal vein Stoll et al. (1998). Catabolism varies with starch digestion rate (Yin et al., 2019) and amino acid form (protein-bound vs. non- bound) (Eugenio et al., 2023). Given the high energetic cost of diverting amino acids from protein accretion, maximizing their efficiency is critical in reduced crude protein (RCP) diets.

Protein is a major cost in poultry diets, primarily supplied by soybean meal for its high crude protein and ideal amino acid (AA) profile. In poultry nutrition, meeting essential AA requirements and providing nitrogen for non-essential AA synthesis is more critical than CP itself (Aviagen, 2014). Well-formulated low-CP diets often require synthetic AA supplementation to prevent imbalances (Tajudeen et al., 2022). Optimizing poultry nutrition through RCP diets is a strategic approach to improving production efficiency, broiler health, and environmental sustainability. These diets generally involve lowering levels of soybean meal, lysine, arginine, threonine, vitamins B and K, dietary cation–anion balance (DCAB), phosphorus, and the phytate-to-total phosphorus ratio, while increasing the inclusion of corn, oil, starch, crystalline essential amino acids, the methionine + cysteine to lysine ratio, and the starch-to-fat ratio. RCP strategies are particularly valuable in developing countries, where reducing dependence on imported soybean meal can lower production costs and environmental burdens (Salahi et al., 2025). Reducing CP intake decreases undigested nitrogen in the hindgut, thereby reducing fermentation and diarrhea and supporting gut morphology, blood protein levels, and growth performance (Gu & Li, 2004). Moreover, reducing dietary soy protein—especially β-conglycinin—has been linked to altered gut short-chain fatty acid (SCFA) profiles and improved cardiac health in mice (Furukawa et al., 2024).

Dietary protein levels impact gut morphology and function, typically assessed via villus height, crypt depth, and surface area (Wang & Peng, 2008). While some studies report reduced performance with low-protein diets (Berres et al., 2010), others find no adverse effects (Widyaratne & Drew, 2011). Laudadio et al. (2012) observed that reducing CP from 22.5 % to 20.5 % increased villus height and the villus height-to-crypt depth ratio (VH/CD) in the duodenum and ileum. Similarly, Barekatain et al. (2019) showed that reducing protein from 20.2 % to 17 % modulated gene expression (↓ZO-2, ↑SGLT1). While downregulation of the tight junction protein ZO-2 can be associated with compromised barrier function, in the context of a well-balanced RCP diet, it may reflect an adaptive metabolic response, suggesting such diets can maintain intestinal health.

Furthermore, dietary vitamins and insoluble fiber are key modulators of intestinal health. Vitamins (e.g., A, D, E, B complex) play crucial roles in epithelial cell differentiation, immune function, and antioxidant defense (Reboul, 2019). Insoluble fiber influences gut motility, digesta passage rate, and can stimulate mucosal morphology and cecal development (Moradi et al., 2021).

Given the intestine's pivotal role in nutrient assimilation and broiler health, a thorough understanding of how dietary interventions impact gut structure is essential. While previous research has assessed the influence of reduced CP on gut morphology, such studies have often been limited in scope. Moreover, the combined effects of lysine restriction, CP reduction, vitamin fortification, and insoluble fiber supplementation on comprehensive intestinal morphometric traits have not been adequately studied through multi-scale microscopic evaluations. . Therefore, this study hypothesizes that dietary reductions in crude protein and lysine, in conjunction with fiber and vitamin modifications, significantly influence intestinal architecture and villus-crypt dynamics. The objective is to provide a comprehensive histo-morphological analysis of the small intestine across multiple magnifications, offering deeper insights into how nutritional strategies reshape gastrointestinal structure in broilers. This need for optimized nutrient matrices in RCP diets is aligned with research emphasizing the critical role of digestible amino acid formulation and lysine levels in supporting broiler performance and health (Nasr & Kheiri, 2017).

## Material and methods

### Broiler management and dietary formulation

A total of 756 one-day-old male Ross 308 broiler chicks were randomly allocated to nine dietary treatments in a completely randomized design (CRD), with six replicates (pens) per treatment and 12 birds/m² (approximately 30 kg/m² final density). Birds had free access to feed and water via bell drinkers and tube feeders per pen. Birds received VITABRON (Newcastle Disease and Infectious Bronchitis) via eye drop at day 7, Avinew Neo (live Newcastle) in drinking water at day 14, and VAXXON® H120-CLONE (Newcastle + Infectious Bronchitis) in drinking water at day 23.

A post-hoc power analysis was conducted using G*Power software (version 3.1.9.7) following Faul et al. (2009) to validate the sample size. Assuming a medium effect size (*f* = 0.25), α = 0.05, and nine treatment groups, the achieved power (1−β) was >0.95. This demonstrates that the chosen design (*n* = 756 birds, including 108 samples for morphometry) provided sufficient sensitivity to detect biologically relevant differences, ensuring robustness and reliability of the results (Faul et al., 2009).

Temperature and other environmental parameters were strictly controlled according to Ross 308 management guidelines. Lighting was initially provided nearly continuously to facilitate feed and water adaptation (23L:1D for days 0–7, 22L:2D for days 8–10, and 20L:4D for days 11–40). Although this regime provided less than the total 6 h of darkness required by the European Union Council Directive 2007/43/EC, it did meet the critical requirement of including at least one uninterrupted 4 h dark period per 24 h after the first week of age. At day 10, birds were weighed collectively (mean 260.9 g) before being assigned to pens. After distribution (54 birds per treatment), post-transfer weights ranged from 252.9 to 257.6 g. Starter-phase feed intake (0–10 days; pelleted) averaged 240 g per bird.

Birds were fed a corn–soybean meal–based diet across three phases (days 10–20, 21–30, and 31–40), incorporating a 12 % reduction in CP. Control diets provided 20.5 %, 19.0 %, and 18.0 % CP across the respective phases, while reduced-CP (RCP) diets delivered 18.0 %, 16.72 %, and 15.9 %. The experimental design was structured as a compensatory matrix to test interactions between key nutrients specifically within this RCP background, rather than to evaluate factors in isolation. Consequently, the factorial arrangement focused on lysine, vitamin, and fiber combinations without including a standalone fiber treatment, prioritizing the identification of synergistic effects under protein-reduced conditions. Digestible lysine (dLys) levels were formulated at a high level based on 6.2 % of dietary crude protein, corresponding to 1.15 %, 1.02 %, and 0.96 %, while low dLys groups (L) were limited to 5.7 % of dietary protein (dLys/CP × 100). All diets maintained ideal amino acid to dLys ratios (Table 1, 2). The RCP diets further varied by inclusion of insoluble fiber (0.5 % Arbocel® RC Fine; F1 vs. F0) and by vitamin level (standard Aviagen® guideline; V1 vs. fortified level; V2) as shown in Table 3. All feed compositions were verified through AMINO® lab analysis. Treatment codes are as follows: H and L denote diets with a digestible lysine-to-crude protein ratio of 6.2 and 5.7 g/100 g, respectively; V1 and V2 represent vitamin supplementation at standard (Aviagen® recommendation) and extra fortification levels, respectively; F0 and F1 indicate insoluble fiber (Arbocel®) supplementation at 0 % and 0.5 %, respectively.

**Table 1:** Ingredients and formulation of the diets fed between days 10–20 and 31–40 days (as-fed basis, % unless noted).

Treatment	Control	HV_1_F_0_	HV_1_F_1_	HV_2_F_0_	HV_2_F_1_	LV_1_F_0_	LV_1_F_1_	LV_2_F_0_	LV_2_F_1_
Ingredients of experimental diets10–20 days (as-fed, %)									
Corn ( %)	58.21	68.86	68.01	68.86	68.01	66.97	66.09	66.97	66.09
Soybean ( %)	34.45	24.52	24.35	24.52	24.35	26.87	27.04	26.87	27.04
Soybean oil ( %)	3.34	1.23	1.62	1.23	1.62	1.87	2.17	1.87	2.17
DCP ( %)	1.62	1.7	1.71	1.7	1.71	1.68	1.68	1.68	1.68
Calcium carbonate	0.68	0.69	0.69	0.69	0.69	0.69	0.69	0.69	0.69
Sodium bicarbonate	0.22	0.37	0.37	0.37	0.37	0.26	0.26	0.26	0.26
Sodium chloride	0.24	0.14	0.14	0.14	0.14	0.21	0.21	0.21	0.21
Potassium carbonate	0	0.08	0.08	0.08	0.08	0	0	0	0
Vit. Min premix	0.5	0.5	0.5	0.5	0.5	0.5	0.5	0.5	0.5
Arbocel® fiber	0	0	0.61	0	0.61	0	0.42	0	0.42
Choline	0	0.04	0.04	0.04	0.04	0.03	0.03	0.03	0.03
Dl-methionine	0.36	0.43	0.43	0.43	0.43	0.32	0.32	0.32	0.32
Lysine-Hcl	0.29	0.55	0.56	0.55	0.56	0.34	0.34	0.34	0.34
L-Thre	0.15	0.27	0.27	0.27	0.27	0.16	0.16	0.16	0.16
L-Val	0.07	0.21	0.22	0.21	0.22	0.08	0.08	0.08	0.08
L-Isoleucine	0.01	0.18	0.18	0.18	0.18	0.05	0.05	0.05	0.05
L-Arg	0.01	0.27	0.28	0.27	0.28	0.09	0.08	0.09	0.08
L-Trp	0	0.02	0.02	0.02	0.02	0	0	0	0
L-Gly	0	0.21	0.2	0.21	0.2	0.044	0.044	0.044	0.044
Sum	100	100	100	100	100	100	100	100	100
Ingredients of experimental diets 31–40days (as-fed, %)									
Corn	66.79	76.08	75	76.08	75	73.91	73.23	73.91	73.23
Soybean	26.71	17.81	18.02	17.81	18.02	20.65	20.78	20.65	20.78
Oil	2.46	0.63	0.99	0.63	0.99	1.31	1.54	1.31	1.54
DCP	1.35	1.42	1.43	1.42	1.43	1.4	1.4	1.4	1.4
Calcium carbonate	0.61	0.62	0.61	0.62	0.61	0.62	0.61	0.62	0.61
Sodium bicarbonate	0.25	0.39	0.39	0.39	0.39	0.28	0.28	0.28	0.28
Sodium chloride	0.22	0.13	0.13	0.13	0.13	0.2	0.2	0.2	0.2
Potassium carbonate	0	0.09	0.09	0.09	0.09	0	0	0	0
Vit. Min premix	0.5	0.5	0.5	0.5	0.5	0.5	0.5	0.5	0.5
Arbocel® fiber	0	0	0.52	0	0.52	0	0.33	0	0.33
Choline	0.01	0.05	0.05	0.05	0.05	0.04	0.04	0.04	0.04
Dl-methionine	0.33	0.4	0.4	0.4	0.4	0.28	0.28	0.28	0.28
Lysine-Hcl	0.34	0.57	0.57	0.57	0.57	0.35	0.35	0.35	0.35
L-Thre	0.15	0.26	0.26	0.26	0.26	0.15	0.15	0.15	0.15
L-Val	0.09	0.22	0.22	0.22	0.22	0.09	0.09	0.09	0.09
L-Isoleucine	0.07	0.22	0.22	0.22	0.22	0.08	0.08	0.08	0.08
L-Arg	0.11	0.35	0.35	0.35	0.35	0.15	0.15	0.15	0.15
L-Trp	0	0.03	0.03	0.03	0.03	0	0	0	0
L-Gly	0	0.23	0.23	0.23	0.23	0.037	0.037	0.037	0.037

Treatment (H: High Lys/CP=6.2 %; L: Low Lys/CP=5.7 %; V1: Standard Vitamin; V2: Enhanced Vitamin; F0: No Fiber; F1: 0.5 % Arbocel® Fiber). Minor deviations (±0.1 %) are attributable to rounding of ingredient inclusion rates. All ingredients are expressed as percentage of diet (as-fed basis), equivalent to g/kg. Soybean oil was used as the dietary fat source.

**Table 2:** Formulated and analyzed nutrient composition of the experimental diets during 10–40 days of age ( %).

Treatment	Control	HV_1_F_0_	HV_1_F_1_	HV_2_F_0_	HV_2_F_1_	LV_1_F_0_	LV_1_F_1_	LV_2_F_0_	LV_2_F_1_
Calculated nutrient composition ( %) 10–20 days									
ME (kcal/kg)	3050	3050	3050	3050	3050	3050	3050	3050	3050
CP	20.5*	18	18	18	18	18	18	18	18
CP (Analyzed)	21.58*	18.71	18	18.7	17.99	18.82	18.95	18.81	18.92
Starch/ CP	1.803	2.429	2.399	2.429	2.399	2.363	2.332	2.363	2.332
Lys (SID)	1.144	1.143	1.143	1.143	1.143	1.026	1.026	1.026	1.026
*M* + *C*(SID)	0.892	0.892	0.892	0.892	0.892	0.8	0.8	0.8	0.8
Thr (SID)	0.766	0.766	0.766	0.766	0.766	0.687	0.687	0.687	0.687
Arg (SID)	1.19	1.189	1.189	1.189	1.189	1.067	1.067	1.067	1.067
Tryp (SID)	0.212	0.212	0.212	0.212	0.212	0.176	0.176	0.176	0.176
Ca	0.756	0.756	0.756	0.756	0.756	0.756	0.756	0.756	0.756
AV. P	0.42	0.42	0.42	0.42	0.42	0.42	0.42	0.42	0.42
Phos /CP× 100	2.05	2.33	2.33	2.33	2.33	2.33	2.33	2.33	2.33
( %)Sodium	0.18	0.18	0.18	0.18	0.18	0.18	0.18	0.18	0.18
( %)Potassium	0.872	0.75	0.75	0.75	0.75	0.75	0.75	0.75	0.75
DCAB (mEq/kg)	224.4	189.8	189.8	189.8	189.8	194.1	194.1	194.1	194.1
Calculated nutrient composition ( %) 20–30 days									
ME (kcal/kg)	3075	3075	3075	3075	3075	3075	3075	3075	3075
CP	19	16.72	16.72	16.72	16.72	16.72	16.72	16.72	16.72
CP (Analyzed)	19.26	16.43	16.29	16.71	16.34	17.43	16.6	16.32	16.73
Starch/ CP	2.116	2.786	2.743	2.786	2.743	2.705	2.674	2.705	2.674
Lys (SID)	1.062	1.062	1.062	1.062	1.062	0.953	0.953	0.953	0.953
Met (SID)	0.58	0.612	0.613	0.612	0.613	0.517	0.517	0.517	0.517
*M* + *C*(SID)	0.828	0.828	0.828	0.828	0.828	0.743	0.743	0.743	0.743
Thr (SID)	0.711	0.711	0.711	0.711	0.711	0.639	0.639	0.639	0.639
Arg (SID)	1.125	1.125	1.125	1.125	1.125	1.01	1.01	1.01	1.01
Gly+Ser (SID)	1.455	1.455	1.455	1.455	1.455	1.258	1.258	1.258	1.258
Tryp (SID)	0.19	0.19	0.19	0.19	0.19	0.158	0.158	0.158	0.158
Ca	0.72	0.72	0.72	0.72	0.72	0.72	0.72	0.72	0.72
AV. P	0.4	0.4	0.4	0.4	0.4	0.4	0.4	0.4	0.4
Phos /CP× 100	2.11	2.39	2.39	2.39	2.39	2.39	2.39	2.39	2.39
Calculated nutrient composition ( %) 30–40 days									
ME (kcal/kg)	3100	3100	3100	3100	3100	3100	3100	3100	3100
CP	18	15.9	15.9	15.9	15.9	15.9	15.9	15.9	15.9
CP (Analyzed)	18.88	17.02	15.68	15.66	16.15	15.62	15.68	16.62	16.83
Starch/ CP	2.356	3.038	2.995	3.038	2.995	2.952	2.925	2.952	2.925
Lys (SID)	1.019	1.021	1.021	1.021	1.021	0.906	0.906	0.906	0.906
Met (SID)	0.576	0.61	0.61	0.61	0.61	0.507	0.507	0.507	0.507
*M* + *C*(SID)	0.817	0.817	0.817	0.817	0.817	0.725	0.725	0.725	0.725
Thr (SID)	0.683	0.684	0.684	0.684	0.684	0.607	0.607	0.607	0.607
Arg (SID)	1.09	1.092	1.092	1.092	1.092	0.97	0.97	0.97	0.97
Gly+Ser (SID)	1.362	1.362	1.362	1.362	1.362	1.187	1.187	1.187	1.187
Tryp (SID)	0.175	0.175	0.175	0.175	0.175	0.147	0.147	0.147	0.147
Ca	0.65	0.65	0.65	0.65	0.65	0.65	0.65	0.65	0.65
AV.P	0.36	0.36	0.36	0.36	0.36	0.36	0.36	0.36	0.36
Ca / Phos %	1.8	1.8	1.8	1.8	1.8	1.8	1.8	1.8	1.8
Phos /CP× 100	2	2.64	2.64	2.64	2.64	2.64	2.64	2.64	2.64
Na ( %)	0.18	0.18	0.18	0.18	0.18	0.18	0.18	0.18	0.18
K ( %)	0.749	0.653	0.653	0.653	0.653	0.653	0.653	0.653	0.653
DEB (mEq/kg)	193	168.4	168.5	168.4	168.5	168.7	168.8	168.7	168.8

⁎ The discrepancy between calculated and analyzed CP (e.g., 20.5 % vs. 21.58 %) arises from natural variations in ingredient composition; analyses were conducted post-formulation. CP was reduced by ∼12 % compared to the control to test the sustainability of reduced-crude protein (RCP) diets, following Salahi et al. (2025). Lysine was isolated as an independent factor (*H* = 6.2, *L* = 5.7 g dLys/100 g CP) to examine its specific role in AA balance, despite proportional reductions in other AAs. The Starch/CP and Phos/CP×100 ratios are reported to highlight shifts in energy-protein density and nutrient utilization in RCP diets. Abbreviations: SID: Standardized ileal digestible; AV. P: Available phosphorus; DCAB/DEB: Dietary cation-anion balance.

**Table 3:** Dietary vitamin supplementation levels: standard (Aviagen®) recommendations (V1) versus extra fortification (V2).

Vitamins	Unit	Recommendations level (V1)		Extra supplementation level (V2)	
		Grower	Finisher	Grower	Finisher
A	IU/kg	11,000	10,000	13,000	15,000
B_1_	mg/kg	4	3	5	6
B_2_	mg/kg	8	7	15	20
B_3_	mg/kg	65	50	65	70
B_5_	mg/kg	20	15	20	25
B_6_	mg/kg	4	3	5	8
B_9_	mg/kg	2	1.8	3	5
B_12_	mg/kg	0.018	0.016	0.05	0.07
D_3_	IU/kg	4500	4000	4500	5000
E	IU/kg	65	55	100	130
K_3_	mg/kg	3.6	3.2	6	9
H_2_	mg/kg	0.28	0.22	0.3	0.6

Vitamin Fortification Rationale: The vitamin levels in the V2 treatments were elevated by 20–50 % above the Aviagen® recommendations. This decision was based on pilot study data indicating benefits in supporting gut health under reduced-crude-protein (RCP) diet-induced stress. The greater increase in the finisher phase was applied to compensate for the age-related decline in feed intake efficiency and to address the specific maintenance and health-support needs of older birds. Treatment Codes: H/L: digestible lysine-to-crude protein ratio of 6.2/5.7 g/100 g; V1/V2 (coded as S/E in tables): standard/enhanced vitamin levels; F0/F1: 0 %/0.5 % insoluble fiber (Arbocel®). Abbreviations for Vitamins: B1 (thiamine), B2 (riboflavin), B3 (niacin), B5 (pantothenic acid or calpan), B6 (pyridoxine), B₉ (folate), B12 (cobalamin), D3 (cholecalciferol), K3 (menadione), H2 (biotin).

### Intestinal morphometric assessment:

All experimental procedures were approved by the University Animal Care and Use Committee (Protocol No: ZNU-AEC-2023–015). At the end of the experiment (day 40), two broilers per replicate (*n* = 108) were randomly selected and birds were slaughtered in accordance with the Institutional Animal Care and Use Committee (IACUC) guidelines and the recommendations of the American Veterinary Medical Association for the euthanasia of poultry (AVMA, 2020). After a fasting period of 4–5 h, birds were humanely euthanized by cervical dislocation, followed immediately by exsanguination via jugular vein severance, to minimize distress and ensure rapid death before organ collection. The entire small intestine—including the duodenum (from the pyloric junction to the end of the pancreatic loop), jejunum (from the end of the duodenal loop to Meckel’s diverticulum), and ileum (from Meckel’s diverticulum to the ileocecal junction**)—**as well as both ceca, were excised. Intestinal segments were emptied of contents by gentle manipulation and rinsed with 0.85 % saline solution without applying pressure to preserve tissue integrity.

All segments were weighed using a precision digital scale (±0.1 g), and expressed as % of body weight where appropriate (Table 4), and lengths were measured with a standardized ruler. The ratio of body weight (g) to the total intestinal weight and length (g and cm, respectively) was calculated to assess intestinal development (Table 4). To estimate cecal surface area (CSA, cm²)—a functional parameter related to nutrient absorption and microbial fermentation—the cecal diameter was measured at three anatomically defined locations: proximal (near the ileocecal junction), middle, and distal regions. The mean diameter (typically ranging from 0.8 to 1.0 cm) was used in the formula:

**Table 4:** Morphometric parameters of gastrointestinal organs in broilers at 40 days of age fed reduced-crude protein diets with different lysine ratios, vitamin, and insoluble fiber levels.

Treatment	DeW	DeL (cm)	JejW	JejL	IleW (g)	IleL (cm)	SmitW	SmitL	BW/ SmitW	BW/ SmitL	CecW (g)	CecL	CSA (cm^2^)
	(g)		(g)	(cm)			(g)	(cm)	(g/g)	(g/cm)		(cm)	
Control	22.52	13.41	38.86	79.33^ab^	34.35	76.33	95.73	169.9	29.35	16.54	14.25	17.6	47.53
HV_1_F_0_	21.48	12.9	35.5	78.50^ab^	29.04	69.9	71.68	134.4	30.93	16.46	13.92	16.9	38.03
HV_1_F_1_	23.48	13.18	38.14	80.36^ab^	35.97	70.09	89.47	150.1	27.97	16.89	15.92	19.13	49.57
HV_2_F_0_	21.51	13.4	37.54	83.11^ab^	32	70	68.29	124.8	29.75	16.21	14.36	18.11	36.68
HV_2_F_1_	20.92	13.25	35.8	72.00^b^	33.03	71.25	89.75	156.5	30.26	17.21	11.83	17.08	48.27
LV_1_F_0_	21.46	12.91	36.11	79.36^ab^	35.1	72.36	84.95	150.9	29.02	16.43	15.8	17.73	43.88
LV_1_F_1_	23.75	14.5	37.4	85.81^a^	35.62	75.27	88.71	160.9	28.91	16.03	15.47	18.18	47.1
LV_2_F_0_	23.2	12.83	41.42	77.75^ab^	34.84	72.25	99.46	162.8	28.05	17.26	15.7	17.36	46.9
LV_2_F_1_	22.11	13.63	34.75	72.63^b^	32.21	75.72	81.65	148.5	30.65	16.75	14.42	16.91	43.8
SEM	1.43	0.715	2.55	3.89	2.56	5.42	11.24	20.02	1.48	0.996	1.49	1.34	5.39
P-Value*	0.384	0.231	0.201	0.011	0.22	0.595	0.139	0.473	0.524	0.924	0.14	0.82	0.348
Lys	0.218	0.392	0.551	0.832	0.123	0.15	0.144	0.197	0.428	0.903	0.09	0.803	0.51
Vit	0.439	0.766	0.655	0.017	0.411	0.998	0.842	0.922	0.478	0.344	0.086	0.343	0.834
Fiber	0.279	0.064	0.33	0.44	0.291	0.398	0.174	0.162	0.815	0.762	0.407	0.44	0.032
Lys×Vit	0.497	0.319	0.503	0.165	0.594	0.935	0.807	0.906	0.104	0.64	0.305	0.743	0.945
Lys×Fiber	0.923	0.099	0.24	0.16	0.047	0.998	0.029	0.249	0.743	0.248	0.709	0.583	0.094
Vit×Fiber	0.043	0.457	0.037	0.003	0.161	0.645	0.403	0.771	0.113	0.973	0.146	0.176	0.582
Lys×Vit×Fiber	0.748	0.663	0.39	0.959	0.743	0.757	0.325	0.383	0.979	0.954	0.381	0.445	0.736

Data are presented as least squares means. Within a column, means without a common superscript letter differ significantly (*P* < 0.05). SEM: Standard error of the mean. Abbreviations: DeW: Duodenum weight; DeL: Duodenum length; JejW: Jejunum weight; JejL: Jejunum length; IleW: Ileum weight; IleL: Ileum length; SmitW: Small intestine weight; SmitL: Small intestine length; BW/SmitW: Body weight to small intestine weight ratio; BW/SmitL: Body weight to small intestine length ratio; CecW: Total cecal weight (sum of both ceca); CecL: Average cecal length; CSA: Cecal surface area (calculated as 2πr × *L*, where *r* = CecW/[2×π×CecL]). Treatment codes: H and L denote digestible lysine-to-crude protein ratios of 6.2 and 5.7 g/100 g, respectively. V1 and V2 represent vitamin supplementation at standard (Aviagen® recommendation) and extra fortification levels, respectively. F0 and F1 indicate insoluble fiber supplementation at 0 % and 0.5 % (Arbocel®), respectively. Data are least-squares means ± SEM (*n* = 12 birds/replicate). P-values from 2 × 2 × 2 factorial ANOVA.

⁎ Overall P-Value.

CSA = 2πr × *L*, where r is half of the mean diameter, and L is the measured length of the cecum. This approach provides a reliable and physiologically relevant estimate of cecal surface morphology.

### Jejunal histomorphometry and tissue processing

**Fig. 1:**
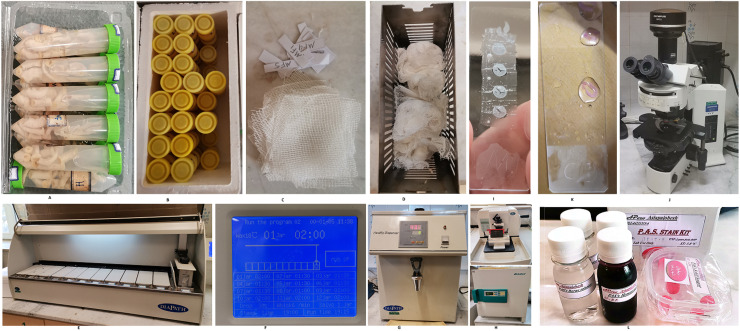
**Workflow of jejunal tissue processing and staining for histomorphometric analysis.** (A–C) Fixation in 10 % neutral buffered formalin. (D–F) Paraffin embedding and block preparation. (G–I) Microtome sectioning and slide mounting. (J–L) Periodic acid–Schiff (PAS) staining kit and the Olympus BX51 light microscope used for image acquisition.

Segments (2 to 3 cm) from the mid-jejunum were excised and fixed in 10 % neutral buffered formalin free of precipitates for 24 h. The formalin was then renewed, and samples were fixed for an additional 72 h before being refrigerated until histological processing. Small jejunal tissue samples from each replicate were excised using a scalpel and processed with a DiAPATH® automated tissue processor. To prevent tissue shrinkage, water was removed by passing samples through graded ethanol concentrations. Samples, wrapped in sterile gauze, were sequentially immersed in jars containing distilled water, ascending ethanol concentrations (70 %, 80 %, 96 %, and absolute ethanol [or 98.8 %] in three jars), followed by two jars of pure xylene, and two paraffin infiltration stages at 60 °C. Exposure times for each step were 2, 1.5, 1.5, 1.5, 1.5, 1.5, 1.5, 1, 1, 2, and 4 h, respectively, totaling 19 h. Immediately after processing, tissues were placed at the base of aluminum molds and embedded in high-quality Bio-Optica® bioplast paraffin at 58 °C. Once cooled, the paraffin blocks were stored at refrigerated temperatures. Using a microtome, blocks were sectioned into 5–6 μm-thick slices. Sections were floated on a 54 °C water bath for 20–30 min to remove wrinkles. Using a syringe needle, sections were carefully transferred onto albumin-coated microscope slides and subsequently dried in an electric oven at 54–55 °C for 2 h (Fig. 1).

The dried slides (Lam) were sequentially processed for hematoxylin and eosin (H&E) staining by immersion in xylene I and II (dimethylbenzene, C₈H₁₀), followed by graded ethanol solutions at 99 %, 96 %, and 80 %, and finally rinsed in distilled water. Slides were then stained with hematoxylin, rinsed in distilled water until the excess stain was removed, and counterstained with Eosin Yellowish (Certistain®, Merck KGaA), followed by a brief rinse. Subsequently, the slides were dehydrated through absolute ethanol and cleared in xylene I and II with immersion times of 15, 15, 5, 5, and 5 min; 10 s; 3–4 min; 10 s; 10 min; 10 s; 3 min; and two consecutive 20-minute incubations, respectively. Coverslips (lamellae or cover glass) were mounted using Entellan® mounting medium, ensuring air bubbles were carefully removed prior to sealing.

**Fig. 2:**
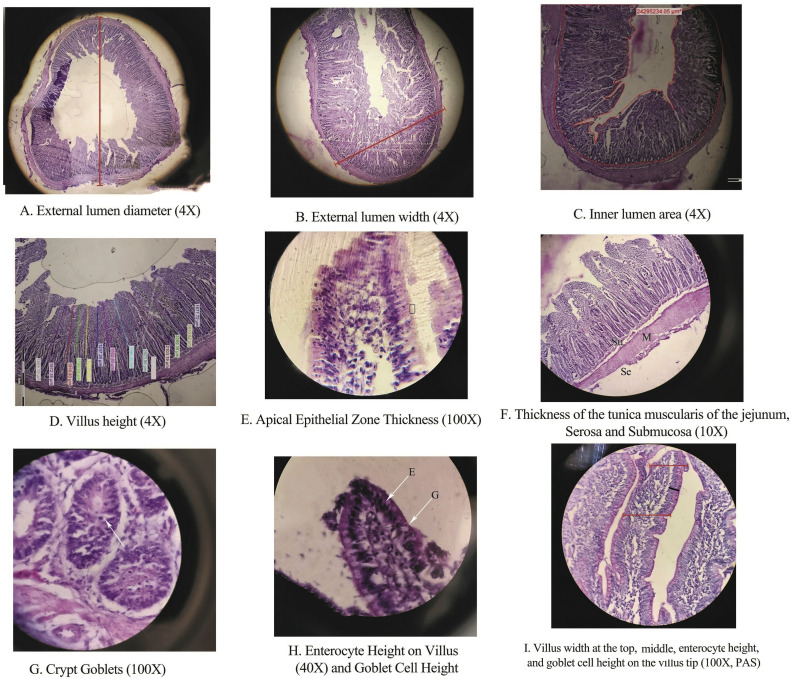
**Representative photomicrographs illustrating morphometric measurements in the jejunum.** (A–C) Low-magnification view (4× objective) showing the jejunal architecture and lumen dimensions. (D) Measurement of villus height (VH) and crypt depth (CD). (E) High-magnification view (100× oil immersion) for measuring apical epithelial zone thickness (AEZ_Th, arrow). (F) Jejunal wall layer thicknesses (serosa, muscularis, submucosa) at 10× magnification. (G) Goblet cell distribution within a crypt (100×). (H) Enterocyte and goblet cell height measurements on a villus (40×, PAS staining). (I) Villus width measurement (100×). Corresponding quantitative data for all treatments, including standard errors (SEM) and significant differences, are presented in Tables 5, 7, 8, and 9.

Microscopic evaluation was performed using an Olympus BX51 light microscope equipped with a DP72 camera and analyzed through Cell^A software at magnifications of 4x, 10x, 40x, and 100x to quantify intestinal morphological parameters as detailed in Table 5, Table 6, Table 7, Table 8. Initial measurements at the 4Xobjective (40Xtotal magnification) included external lumen metrics—width, diameter, area, and perimeter—as well as internal lumen dimensions from crypt base to villus tip. Concurrently, villus length, crypt depth, and villus width were recorded. At 10X objective (100 X total magnification), crypt counts within the lumen and thicknesses of the submucosa, muscularis mucosa, and serosa were assessed. Using the 40X objective (400 x total magnification), goblet cells per crypt, villus width, and crypt morphometrics—length, width, area, and perimeter—were quantified. Crypt area and perimeter were measured by tracing the crypt outline using Cell^A's polygon tool, averaging 10 crypts per section to account for irregularity. The apical epithelial zone thickness (AEZ_Th) was measured under a 100× oil immersion lens (total magnification: 1000) on hematoxylin-stained sections; this included the entire apical portion of epithelial cells (microvilli, cytoplasmic region, and possibly intercellular junctions) (Fig. 2). Also, goblet cell dimensions (length, width, area, and perimeter) in both crypt and villus regions were measured. Finally, sections of villus tips (200 µm in length at 4× magnification or 5000 µm at 100× magnification) were examined to calculate goblet cell number, individual goblet cell area, and the ratio of cumulative goblet cell area to villus tip area. Villus surface area (VSA, mm²) was computed following Sakamoto et al. (2000) as VSA (mm^2^) = 2π × (villus width/2) × villus length, with villus height-to-crypt depth ratio (VH/CD) also calculated to characterize mucosal architecture comprehensively. At 10X magnification, all crypts within the lumen were counted under the microscope to determine the number of crypts per lumen for each replicate. Additionally, at 40X magnification, the number of goblet cells per crypt and the total number of goblet cells across all observable crypts within the field of view were carefully counted for each replicate.

**Table 5:** Jejunal lumen, villus, and crypt morphometric parameters in broilers fed reduced-crude protein diets with varying lysine, vitamin, and fiber levels.

Treatment	Lys	Vit	Fib	OLW (µm)	ILW (µm)	OLD (µm)	ILD (µm)	ILA (µm^2^)	ILP (µm)	EpiLen (µm)	VH (µm)	VW (µm)	CD (µm)	VH/CD	VSA (mm^2^)
Control (20.5 % CP)	-	-	-	5978.8	4976.3	14,861.6	13,206.49	59,642,971	116,254	2196.7^a^	1927.6^a^	304.1	268.8	7.19	1.83^ab^
HV_1_F_0_	6.2	S	0	5822.6	5124.5	19,200.1	17,820.9	71,853,556	110,975	2041.2^ab^	1765.6^abc^	251.5	275.1	6.43	1.402^c^
HV_1_F_1_	6.2	S	1	4628.7	3635.9	15,973.6	12,927.8	57,636,280	81,317	1914.7^b^	1640.2^b^	249.5	274.5	6.32	1.50^bc^
HV_2_F_0_	6.2	E	0	4395.6	3369.9	18,190.4	16,831.1	54,417,928	91,025	2162.2^a^	1888.1^ab^	268.1	274.1	6.90	1.59^abc^
HV_2_F_1_	6.2	E	1	5939.3	5289.1	15,095.6	12,765.8	49,806,800	42,525	2028.6^ab^	1769.2^abc^	259.5	261.2	6.69	1.27^c^
LV_1_F_0_	5.7	S	0	4132.8	3147.6	18,400.9	17,232.1	63,920,289	65,652	1981.1^ab^	1688.5^bc^	302.5	293.1	5.76	1.60^abc^
LV_1_F_1_	5.7	S	1	4002.1	3249.8	17,042.6	15,440.9	63,179,749	75,616	2055.1^ab^	1800.2^abc^	238.8	254.5	7.32	1.44^bc^
LV_2_F_0_	5.7	E	0	5032.7	4519.8	20,607.4	17,277.6	64,756,298	79,287	2129.5^ab^	1858.5^abc^	278.1	271.3	7.21	1.98^a^
LV_2_F_1_	5.7	E	1	4185.4	3489.8	16,287.2	13,348.8	51,453,350	79,570	2047.2^ab^	1779.2^abc^	274.8	267.8	6.76	1.43^bc^
SEM				1102.3	1146.2	2709.2	2878.3	11,912,752	29,809	58.1	58.7	24.6	21.3	0.48	0.088
P-Value*				0.51	0.49	0.67	0.78	0.77	0.37	0.007	0.001	0.106	0.421	0.237	0.002
Lys				0.151	0.2	0.672	0.678	0.543	0.377	0.476	0.721	0.207	0.216	0.488	0.246
Vit				0.691	0.528	0.956	0.748	0.307	0.333	0.006	0.005	0.497	0.764	0.260	0.245
Fiber				0.694	0.75	0.188	0.182	0.155	0.157	0.011	0.019	0.103	0.497	0.836	0.001
Lys×Vit				0.828	0.637	0.724	0.953	0.359	0.113	0.767	0.668	0.678	0.612	0.794	0.109
Lys×Fiber				0.483	0.493	0.966	0.645	0.847	0.107	0.537	0.550	0.259	0.981	0.777	0.050
Vit×Fiber				0.479	0.406	0.739	0.918	0.534	0.723	0.045	0.042	0.244	0.806	0.133	0.004
Lys×Vit×Fiber				0.165	0.07	0.711	0.765	0.37	0.921	0.501	0.380	0.092	0.419	0.277	0.917

Data are presented as least squares means. Within a column, means without a common superscript letter differ significantly (*P* < 0.05). SEM: Standard error of the mean. Abbreviations: OLW: Outer lumen width; ILW: Inner lumen width; OLD: Outer lumen diameter; ILD: Inner lumen diameter; ILA: Inner lumen area; ILP: Inner lumen perimeter; EpiLen: Epithelium length (VH+CD); VH: Villus height; VW: Villus width; CD: Crypt depth; VH/CD: Villus height to crypt depth ratio; VSA: Villus surface area [VSA = 2π × (VW/2) × VH]. S: Standard vitamin level (Aviagen recommendation), E: Enhanced vitamin level. Data are least-squares means ± SEM (*n* = 12 birds/replicate). P-values from 2 × 2 × 2 factorial ANOVA.

⁎ Overall P-Value.

**Table 6:** AI-assisted morphometric analysis of jejunal villi and crypts (4× magnification) in broilers under different dietary regimens.

Treatment	Lys	Vit	Fib	VN	VH (µm)	VWtp (µm)	VWm (µm)	CD (µm)	VH/CD
Control	-	-	-	13.53	1806.6^a^	198.5	237.9	285.2	6.02
HV_1_F_0_	6.2	S	0	14.55	1641.1^b^	207.8	247.9	289.8	5.38
HV_1_F_1_	6.2	S	1	15.51	1718.3^ab^	207.5	253.5	294.5	5.55
HV_2_F_0_	6.2	E	0	15.05	1731.4^ab^	207.8	250.4	292.6	5.62
HV_2_F_1_	6.2	E	1	15.96	1706.6^ab^	207.9	251.1	290.6	5.54
LV_1_F_0_	5.7	S	0	14.15	1668.3^b^	200.9	244.1	286.5	5.49
LV_1_F_1_	5.7	S	1	14.73	1689.3^ab^	203.5	248.3	289.2	5.54
LV_2_F_0_	5.7	E	0	14.82	1687.1^b^	204.3	249.1	292.5	5.48
LV_2_F_1_	5.7	E	1	13.93	1662.3^b^	198.4	243.2	285.3	5.55
SEM				0.84	36.9	4.55	5.55	4.43	0.060
P-Value*				0.473	0.049	0.376	0.433	0.684	0.308
Lys				0.148	0.246	0.076	0.276	0.271	0.995
Vit				0.708	0.310	0.920	0.994	0.950	0.184
Fiber				0.533	0.993	0.795	0.806	0.879	0.291
Lys×Vit				0.626	0.282	0.862	0.998	0.773	0.158
Lys×Fiber				0.350	0.554	0.811	0.623	0.388	0.572
Vit×Fiber				0.496	0.045	0.509	0.345	0.264	0.117
Lys×Vit×Fiber				0.525	0.411	0.479	0.751	0.540	0.089

Data are presented as least squares means. Within a column, means without a common superscript letter differ significantly (*P* < 0.05). SEM: Standard error of the mean. Abbreviations: VN: Villus number per field; VH: Villus height; VWtp: Villus width at the top; VWm: Villus width at the middle; CD: Crypt depth; VH/CD: Villus height to crypt depth ratio. S: Standard vitamin level (Aviagen recommendation), E: Enhanced vitamin level. Data are least-squares means ± SEM (*n* = 12 birds/replicate). P-values from 2 × 2 × 2 factorial ANOVA.

⁎ Overall P-Value.

**Table 7:** Thickness of jejunal wall layers and crypt number at 10× magnification: a comparison between H&E and PAS staining methods.

Treatment	Lys	Vit	Fib	H&E Staining (Cell^A Software)					PAS Staining (ImageJ Software)			
				LuCrptNo	SeroTh (µm)	MuslTh (µm)	SubmuTh (µm)	TotalTh (µm)	SeroTh (µm)	MuslTh (µm)	SubmuTh (µm)	TotalTh (µm)
Control	-	-	-	286.7	137.9^ab^	547.9^ab^	206.4^ab^	896.3^a^	93.6	370.1	206.7	670.9
HV_1_F_0_	6.2	S	0	312.7	150.2^a^	476.8^abc^	204.2^ab^	776.6^ab^	98.6	385.1	216.8	700.6
HV_1_F_1_	6.2	S	1	168.3	86.5^b^	405.4^bc^	187.5^ab^	712.5^ab^	98.7	389.0	219.3	707.1
HV_2_F_0_	6.2	E	0	271.2	153.1^a^	585.5^a^	203.8^ab^	870.6^a^	99.9	389.6	219.8	709.4
HV_2_F_1_	6.2	E	1	270.8	110.7^ab^	345.1^c^	168.5^ab^	567.5^b^	99.5	389.6	219.7	709.1
LV_1_F_0_	5.7	S	0	217.3	140.2^ab^	382.5^bc^	197.7^ab^	720.2^ab^	94.4	373.9	209.3	677.6
LV_1_F_1_	5.7	S	1	228.4	119.8^ab^	339.7^c^	179.1^ab^	692.4^ab^	96.6	383.5	215.6	695.8
LV_2_F_0_	5.7	E	0	177.3	113.6^ab^	370.2^bc^	155.4^b^	639.2^ab^	96.9	384.9	216.6	698.4
LV_2_F_1_	5.7	E	1	211.9	155.9^a^	485.9^abc^	227.5^a^	787.3^ab^	94.1	376.4	210.9	681.3
SEM				29.8	14.83	43.22	16.64	65.84	2.42	8.52	5.95	15.33
P-Value*				0.259	0.04	0.017	0.043	0.001	0.466	0.514	0.293	0.506
Lys				0.002	0.872	0.058	0.755	0.651	0.053	0.118	0.118	0.102
Vit				0.749	0.630	0.289	0.968	0.944	0.781	0.658	0.657	0.677
Fiber				0.047	0.048	0.056	0.981	0.293	0.895	0.831	0.833	0.876
Lys×Vit				0.024	0.320	0.996	0.706	0.868	0.757	0.952	0.925	0.918
Lys×Fiber				0.023	0.088	0.092	0.348	0.059	0.966	0.896	0.896	0.907
Vit×Fiber				0.006	0.250	0.560	0.434	0.763	0.423	0.293	0.294	0.310
Lys×Vit×Fiber				0.035	0.594	0.044	0.012	0.045	0.505	0.491	0.490	0.491

Data are presented as least squares means. Within a column, means without a common superscript letter differ significantly (*P* < 0.05). SEM: Standard error of the mean. Abbreviations: LuCrptNo: Number of crypts surrounding the lumen; SeroTh: Serosa thickness; MuslTh: Muscularis propria thickness; SubmuTh: Submucosa thickness; TotalTh: Total wall thickness (sum of Serosa, Muscularis, and Submucosa). S: Standard vitamin level (Aviagen recommendation), E: Enhanced vitamin level. Data are least-squares means ± SEM (*n* = 12 birds/replicate). P-values from 2 × 2 × 2 factorial ANOVA.

⁎ Overall P-Value.

**Table 8:** High-magnification analysis of jejunal crypt dimensions and goblet cell characteristics.

Treatment	40X					100X						
	Gob_CR (No)	CR_Len (µm)	CR_BsWd (µm)	CrArea (µm^2^)	CR_Prm (µm)	AEZ_Th (µm)	CrArea (µm^2^)	CrGbN (Cells)	CrGbAr (µm^2^)	GbCrRt	GbVLH (Cell)	GbVLRt
Control	29.1	3447^a^	1025	3423220^a^	10,007.8^a^	243.1	9224000^a^	31	228200^a^	3.36	22.41	10.05
HV_1_F_0_	26.2	2488.6^ab^	1254	3011932^ab^	7352.7^ab^	231.2	6986000^ab^	26	161259^ab^	3.12	22.22	8.82
HV_1_F_1_	16.6	1883.3^b^	1110	1363333^b^	6075.1^b^	197.5	4565000^b^	17.5	172900^ab^	3.8	20.78	9.83
HV_2_F_0_	25.4	2950.1^ab^	1332	3332837^a^	8181.2^ab^	236.8	8265000^ab^	28.2	202616^ab^	3.52	23.72	10.51
HV_2_F_1_	17.1	2468.6^ab^	961	1833944^ab^	6803.6^ab^	197.4	5395000^ab^	19.5	222950^a^	4.78	22.78	11.68
LV_1_F_0_	14.6	2449.8^ab^	1094	1963478^ab^	7049.8^ab^	208.1	5416000^ab^	17.2	116925^ab^	2.96	18.77	7.03
LV_1_F_1_	13.6	2828.6^ab^	1342	3030100^a^	7608.1^ab^	230.2	8077500^ab^	17.1	187666^ab^	3.82	19.78	11.51
LV_2_F_0_	16.1	2950^ab^	1106	2333433^ab^	8633.3^ab^	227.1	5167000^b^	20	90000^b^	2.82	20.78	8.18
LV_2_F_1_	16.4	2194^b^	1030	1926055^ab^	6087.3^a^	212.5	4911000^b^	19.2	160500^ab^	4.64	20.79	11.33
SEM	1.81	241.8	124.2	43,891	925.1	35.12	100,660	1.52	28,856	0.562	1.33	0.838
P-Value*	0.982	0.002	0.102	0.043	0.001	0.893	0.001	0.667	0.004	0.101	0.607	0.161
Lys	0.001	0.966	0.794	0.014	0.098	0.798	0.099	0.001	0.006	0.728	0.015	0.117
Vit	0.29	0.174	0.167	0.566	0.137	0.723	0.231	0.025	0.697	0.113	0.074	0.023
Fiber	0.003	0.001	0.31	0.0001	0.0001	0.384	0.085	0.001	0.026	0.004	0.571	0.001
Lys×Vit	0.667	0.995	0.083	0.615	0.104	0.803	0.017	0.73	0.089	0.749	0.881	0.229
Lys×Fiber	0.004	0.07	0.14	0.0001	0.014	0.18	0.001	0.001	0.104	0.699	0.268	0.006
Vit×Fiber	0.868	0.192	0.589	0.004	0.003	0.494	0.447	0.892	0.684	0.329	0.881	0.631
Lys×Vit×Fiber	0.823	0.006	0.061	0.001	0.0001	0.657	0.098	0.965	0.862	0.734	0.655	0.455

Data are presented as least squares means. Within a column, means without a common superscript letter differ significantly (*P* < 0.05). SEM: Standard error of the mean. Abbreviations: Gob_CR: Number of goblet cells per crypt; CR_Len: Crypt length; CR_BsWd: Crypt base width; CrArea: Crypt cross-sectional area; CR_Prm: Crypt perimeter; AEZ_Th: Apical epithelial zone thickness; CrGbN: Number of goblet cells per crypt (100×); CrGbAr: Total area of goblet cells per crypt; GbCrRt: Ratio of goblet cell area to crypt area; GbVLH: Number of goblet cells in the villus head; GbVLRt: Ratio of goblet cell area to villus head area. Data are least-squares means ± SEM (*n* = 12 birds/replicate). P-values from 2 × 2 × 2 factorial ANOVA. Note: CrArea represents perimeter-based polygon area (40X: complete crypt outline, *n* = 10 crypts/section; 100X: cumulative goblet cell projection area).

⁎ Overall P-Value.

### Protocol for periodic acid-schiff (PAS) staining of intestinal tissue

The PAS staining protocol for intestinal tissue involved deparaffinization by sequential immersion in xylene I (20 min), xylene II (20 min), 98 % ethanol (3 min), and 80 % ethanol (3 min), followed by rehydration in distilled water. Deparaffinized sections were stained using the Asia Pazhouhesh PAS kit, specifically targeting carbohydrates, glycogen, and mucins. Initially, periodic acid (10 min) was applied to oxidize glycans, followed by rinsing in distilled water, and then incubation with Schiff’s reagent (15 min) to form magenta-colored complexes. After another rinse, a resting period of 10 min was employed to enhance glycogen visualization, followed by brief borax treatment (15 *sec*). Nuclei were counterstained with hematoxylin (20–45 *sec*), with subsequent borax enhancement (15 *sec*), and rinsing. All steps involving photosensitive reagents were conducted in a darkened environment to prevent degradation. The stained sections were then dehydrated using a graded ethanol series (96 %, 99 %), cleared in xylene (4×), and mounted with Entelan**®**. This protocol effectively differentiates carbohydrate-rich components (stained pink/magenta) and nuclei (stained blue), facilitating precise histopathological evaluation (Fig. 1). In all hematoxylin–xylene and PAS-stained preparations, two representative microscopic fields of view were systematically assessed and quantified for each sample. Although PAS staining was applied for goblet cell identification and mucin visualization, future studies may benefit from the use of Alcian Blue or combined Alcian Blue–PAS (AB–PAS) staining to further discriminate acidic mucins and enhance goblet cell characterization.

### Jejunum image analysis using artificial intelligence

Morphometric data from Cell^A software (Olympus BX51 microscope) were validated using ImageJ v1.53t (NIH, Schneider et al., 2012). Images were calibrated at each magnification (scale bar: 100 μm at 4× = 2.39 pixels/μm; 40× = 0.239 pixels/μm). At 4× magnification, villus height, width, and crypt depth were measured, along with the thicknesses of serosa, muscularis, and submucosa (*n* = 108 sections; scale: 2.39 pixels/μm). PAS-stained jejunal sections captured at 40X were analyzed for villus width, enterocyte count and height, and goblet cell number and height. Enterocytes were identified by their basophilic oval nuclei near the lumen; goblet cells by pale cytoplasm, basal nuclei, and mucin-filled vacuoles. Heights were measured in micrometers using calibrated ImageJ tools, and average counts per 400 µm (scale: 0.239 pixels/μm) field were calculated. In total, 54 jejunal images (one per replicate) were analyzed for treatment group comparison. While standardized 2D morphometry (Cell^A and ImageJ) was applied in this study, it is acknowledged that design-based stereology offers the most rigorous and unbiased estimates of intestinal volume and surface characteristics. Future studies should integrate stereological sampling to strengthen quantitative accuracy (Mouton, 2002).

### Statistical analysis

The experiment followed a completely randomized design (CRD) with nine dietary treatments. Each treatment was replicated six times (pens), with 14 birds per pen initially. The treatments were arranged in an incomplete 2 × 2 × 2 + 1 factorial structure. The additional treatment served as a control diet containing standard lysine levels, no supplemental fiber, and standard vitamin inclusion. Data were analyzed using the general linear model (GLM) procedure in SPSS version 22 (SPSS Inc., Chicago, IL, USA), based on the following statistical model:

$$Y_{\text{ijkl}}=\mu +L_{i}+F_{j}+V_{k}+L\times F_{\text{ij}}+L\times V_{\text{ik}}+F\times V_{\text{jk}}+L\times F\times V_{\text{ijk}}+e_{\text{ijkl}}$$

where L, F, and V represent lysine, fiber, and vitamin effects, respectively, along with their two- and three-way interactions. The incomplete nature of the factorial design was addressed by appropriately specifying the missing cells in the GLM to ensure valid estimation of main effects and interactions. Data were analyzed in two sequential stages. In the first stage, all experimental treatments, including the control, were compared using a completely randomized design (CRD) model, and the resulting overall P-values are reported. In the second stage, factorial effects were evaluated using a 2 × 2 × 2 arrangement consisting of two levels of lysine, two levels of vitamin, and two levels of fiber, and the P-values were reported for the main and interaction effects. Exact P-values were reported. Prior to analysis, assumptions of normality and homogeneity of variances were evaluated using the Shapiro–Wilk and Levene’s tests, respectively. When these assumptions were violated, nonparametric alternatives, such as the Kruskal–Wallis test, were applied. Prior to ANOVA, data normality and homogeneity of variances were confirmed using the Shapiro-Wilk and Levene’s tests, respectively. For variables violating parametric assumptions, Kruskal-Wallis non-parametric tests were applied. Mean comparisons were performed using Tukey’s HSD test, with statistical significance set at *P* < 0.05.

## Results

CP reduction (12 %) and lysine restriction (5.7 vs. 6.2 g/kg of diet, equivalent to 5.7 % vs. 6.2 % of CP) had no significant effects on most intestinal morphometric indices (Table 4; P > 0.05), except for jejunal length, which was influenced by protein level (P = 0.011) and a vitamin × fiber interaction (P = 0.003). Insoluble fiber supplementation (F1) significantly increased cecal surface area (CSA) by approximately 15.2 % compared to non-supplemented groups (F0) (P = 0.032). Significant interactions were noted between lysine × fiber for ileal (P = 0.047) and total intestinal weight (P = 0.029), and vitamin × fiber for duodenal and jejunal metrics (P < 0.05). On slaughter day, small intestine-to-body weight ratios were 3.44 %, 2.98 %, and 3.25 % in the control, high-lysine, and lysine-restricted groups, respectively. Overall growth performance (body weight gain and feed conversion ratio) was not significantly affected by dietary treatments in the broader study (data not shown), indicating that the RCP diets maintained production performance. To provide context for the sample size, a detailed morphometric assessment was performed on 108 jejunal samples, including villus height, crypt depth, epithelial and serosa/muscularis/submucosa thickness, as well as goblet cell and enterocyte counts and dimensions. Full results are reported in Table 5, Table 6, Table 7, Table 8, Table 9.

**Table 9:** Effects of dietary lysine level, vitamin fortification, and insoluble fiber supplementation on enterocyte and goblet cell morphometrics in jejunal villus epithelium of broilers, analyzed using imageJ software (40× Magnification, PAS Staining).

Treatment	Ent_Cnt (Cells)	Ent_HA (µm)	Gob_Cnt (Cells)	GobHt (µm)	Ent_Dns (Cells/µm)	Gob_Dens (Cells/µm)
Control	145^ab^	27.5^ab^	12.5	30^ab^	0.345	0.071
HV_1_F_0_	117.3^ab^	23.5^ab^	10.3	24.7^ab^	0.279	0.051
HV_1_F_1_	124^ab^	24.5^ab^	11.6	27.6^ab^	0.295	0.057
HV_2_F_0_	153.3^a^	29^a^	13	32.3^a^	0.365	0.076
HV_2_F_1_	88.5^ab^	20.6^ab^	8.75	21.7^ab^	0.211	0.033
LV_1_F_0_	94.3^ab^	20.9^ab^	10	21.8^ab^	0.225	0.037
LV_1_F_1_	70.6^b^	17.3^b^	9	17.7^b^	0.168	0.021
LV_2_F_0_	124.4^ab^	25.4^ab^	10.8	27.1^ab^	0.296	0.059
LV_2_F_1_	145^ab^	27.3^ab^	12	29^ab^	0.242	0.069
SEM	18.1	3.11	1.55	3.88	0.059	0.01
P-Value*	0.038	0.049	0.795	0.049	0.452	0.218
Lys	0.209	0.243	0.394	0.164	0.11	0.255
Vit	0.02	0.015	0.203	0.032	0.258	0.026
Fiber	0.371	0.314	0.739	0.432	0.181	0.386
Lys×Vit	0.077	0.12	0.471	0.176	0.52	0.115
Lys×Fiber	0.302	0.471	0.509	0.612	0.965	0.434
Vit×Fiber	0.686	0.669	0.479	0.464	0.313	0.615
Lys×Vit×Fiber	0.404	0.411	0.451	0.399	0.955	0.526

Lys: Lysine, Vit: Vitamin, Fib: Fiber, SEM: The standard error of the mean, Ent_Cnt: Number of Enterocytes in Field, Ent_HA: Average Enterocyte Height on Villus, Gob_Cnt: Number of Goblet Cells in Field, GobHt: Average Goblet Cell Height, Ent_Dns: Enterocyte Density, Gob_Dens: Goblet Cell Density. Treatment description: H and L for dLys/CP ratio of 6.2 and 5.7 respectively; V_1_ and V_2_ for vitamin supplementation level of broiler recommendation and extra supplementation respectively; F_0_ and F_1_ for insoluble fiber supplementation levels of 0 and Arbocel® added diets (on average 0.5 %) respectively. Data are least-squares means ± SEM (*n* = 12 birds/replicate). P-values from 2 × 2 × 2 factorial ANOVA.

⁎ Overall P-Value.

**Fig. 3:**
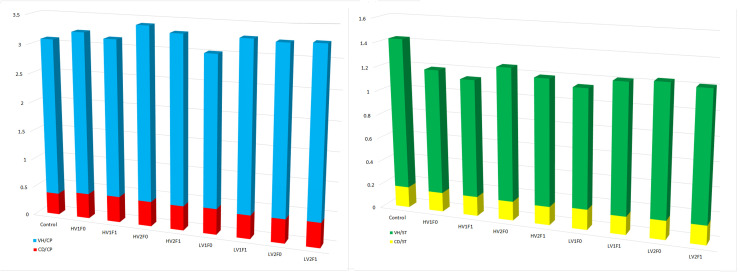
**Graphical representation of villus height relative to nutrient intake.** The ratios of villus height (VH) to crude protein intake (VH/CP) and villus height to starch intake (VH/ST) are presented visually to illustrate comparative relationships between dietary treatments. Please note: These ratios are presented for descriptive, comparative purposes only and are not subject to formal statistical analysis in this context. Their inclusion aims to provide a visual perspective on nutrient utilization efficiency under different dietary regimens. Formal statistical analyses of morphometric parameters are presented in Tables 5–9 and corresponding figures.

Dietary treatments had no significant effect on the gross morphometric parameters of the jejunum, including outer and inner lengths and diameters (*P* > 0.05). However, crude protein (CP) reduction significantly influenced several histomorphological indices, including epithelial length (reduced by 12.3 %, *P* = 0.007), villus height (reduced by 10.5 %, *P* = 0.001), and villus surface area (VSA) (reduced by 18.7 %, *P* = 0.002). Vitamin supplementation significantly affected epithelial length and villus height, whereas the inclusion of insoluble fiber markedly influenced epithelial length, villus height, and VSA (Table 5; *P* < 0.05). Significant lysine × fiber interactions were observed for VSA (*P* = 0.050). Fiber × vitamin interactions also significantly affected villus height (*P* = 0.042), epithelial length (*P* = 0.045), and VSA (*P* = 0.004), indicating a synergistic influence on mucosal architecture. The highest VH/CD ratio (7.32, 7.21) was found in the LV_1_F_1_ and LV2F0 groups, while the control group showed the lowest (7.19). Despite non-significant effects of lysine restriction on gross intestinal structure, marked micro-architectural changes were evident, especially with fiber and vitamin inclusion. As illustrated in Fig. 3, villus height and crypt depth were affected by crude protein and starch intake, along with their intake-adjusted ratios. The left panel shows villus height-to-protein (VH/CP) and crypt depth-to-protein (CD/CP) ratios (µm/g), while the right panel displays villus height-to-starch (VH/ST) and crypt depth-to-starch (CD/ST) ratios.

**Fig. 4:**
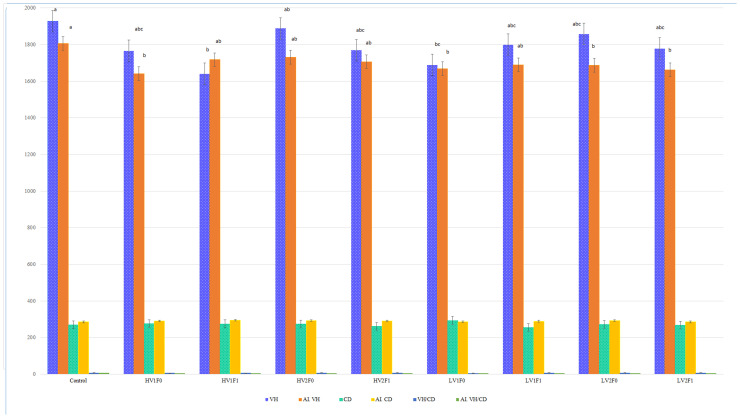
**Comparison of jejunal villus height and crypt depth measurements obtained by two analytical software platforms.** Field-based measurements using Cell^A software (3D cylinder columns) versus image analysis using ImageJ software (patterned box columns). Data are presented as means ± SEM (error bars). For each morphometric parameter (VH or CD), columns without a common**.** Superscript letter differs significantly (*P* < 0.05).

Jejunal villus morphometry was evaluated using two methods. A method-comparison analysis between Cell^A and ImageJ showed high agreement for core morphometric measurements (e.g., villus height, crypt depth). All primary results reported herein are based on Cell^A measurements. (Fig. 4).

Analysis of Table 7 reveals that CP reduction significantly decreased the thickness of the serosa, muscularis, submucosa, and their total (*p* ≤ 0.001), without significantly altering the number of luminal crypts (*p* = 0.259). Lysine restriction significantly affected only crypt number (*p* = 0.002). Fiber supplementation significantly reduced the thickness of the serosa (*p* = 0.048). The greatest total thickness was observed in HV2F0 (870.6 µm) and the control group (896.3 µm), while treatments such as HV1F1 showed reductions, possibly due to altered digestibility under high-lysine and high-fiber conditions. Significant three-way interactions (Lys × Vit × Fib) were observed for crypt number, muscularis, submucosa and their total thickness (*p* ≤ 0.05), indicating complex interplays among dietary factors.

**Fig. 5:**
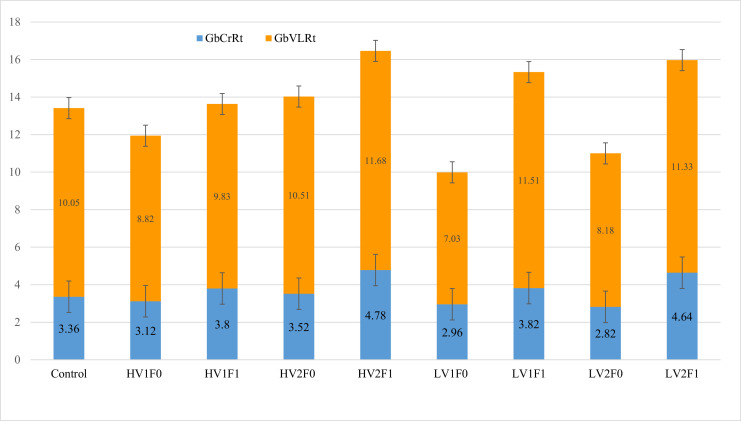
**Impact of dietary treatments on goblet cell area ratios in the jejunum.** Goblet cell area to villus head area ratio (GbVLRt) and goblet cell area to crypt area ratio (GbCrRt) in broilers fed reduced-protein diets with varying lysine (H, L), vitamin (S: standard; E: enhanced), and fiber (F0, F1) levels. The control diet (20.5 % CP) is included for reference. Data are presented as means ± SEM (error bars). Statistical analysis was performed using a 2 × 2 × 2 factorial ANOVA; P-values for main effects and interactions are reported in Table 8. No significant effects of lysine, vitamin, fiber, or their interactions were detected for these ratios (*P* > 0.05).

Morphometric analysis of jejunal crypts and goblet cell distribution at 40X and 100X magnifications revealed significant responses to dietary lysine, vitamin, and insoluble fiber levels. Lysine restriction significantly reduced goblet cell number per crypt (*p* = 0.001) and crypt area (*p* = 0.014), while crude CP reduction led to notable decreases in crypt length (*p* = 0.002), area (*p* = 0.043), and perimeter (*p* = 0.0001). The control group exhibited the largest crypts (3423,220 µm², 40X perimeter area), whereas fiber supplementation markedly suppressed crypt development and goblet cell numbers, particularly in the LV1F1 group (13.6 vs. 29.1, *p* = 0.003) (Table 8). In the control group, crypt goblet cell count (29.1–31 cells; Table 8), Goblet-to-Crypt Ratio (3.36), and Goblet Area-to-Villus Head Area Ratio (10.05) were higher than those in the treatment groups. Goblet cell density was consistently greater in crypts than in villi. Fiber also significantly influenced goblet cell area and increased goblet-to-crypt (*p* = 0.004) and goblet-to-villus head area ratios (*p* = 0.001), suggesting mucosal adaptation. Vitamin effects were modest but significant for crypt goblet number (CrGbN, *p* = 0.025) and Goblet Area to Villi Head Area Ratio (GbVLRt, *p* = 0.023). Significant interactions (Lys×Fiber, Vit×Fiber, Lys×Vit×Fiber; *p* < 0.05) altered crypt area and crypt perimeter, while nutrient levels affected goblet cell ratios (Goblet Area to Villi Head Area Ratio, Goblet to Crypt Ratio), as visualized in Fig. 5.

Statistical analysis based on PAS-stained images analyzed at 40× magnification using ImageJ software (Table 9) showed that CP reduction significantly decreased the number of enterocytes in the field (Ent_Cnt), while lysine restriction had no significant effect on any enterocyte-related parameters. Goblet cell counts remained unaffected. Vitamin fortification had a significant effect on enterocyte count (Ent_Cnt, *p* = 0.02), enterocyte height (Ent_HA, *p* = 0.015), and goblet cell density (Gob_Dens, *p* = 0.026). The HV2F0 group (high lysine with additional vitamins) showed the greatest enterocyte count (153.3 cells) and height (29 µm). Goblet cell height (GobHt) also differed significantly across treatments (*p* = 0.049), reflecting similar trends to enterocyte height. Neither two-way nor three-way interactions significantly influenced any measured parameters, indicating additive rather than synergistic effects of these dietary components on intestinal morphology.

## Discussion

The minimal impact of crude protein (CP) and lysine reduction on gross intestinal dimensions underscores a high degree of adaptive plasticity in the broiler gut. The biologically negligible reduction in jejunal length (maximum 0.84 cm between groups) likely represents an efficient reallocation of digestive resources rather than pathological atrophy, aligning with conserved vertebrate strategies to modulate absorptive surfaces in response to nutrient availability (Karasov, 1996; McWilliams & Karasov, 2014). Importantly, these subtle morphological adaptations occurred without substantial compromise to overall growth performance (body weight gain and feed conversion ratio), suggesting a functional resilience of the gastrointestinal tract under the dietary conditions tested. . The high statistical power achieved (>0.95) supports the reliability of these non-significant findings, indicating that the absence of major treatment effects is unlikely to be due to insufficient sample size (Faul et al., 2009).

The maximal difference in jejunal length between any treatment group and the control was 0.64 cm, representing a biologically negligible variation of <1 %. These findings align with Song et al. (2023), who showed that a 1 % CP reduction with 50–100 g/ton protease had no negative effect on small or large intestine weights. Dietary fiber significantly increased cecal surface area (CSA) (*P* = 0.032), with notable lysine × fiber interactions for ileum (*P* = 0.047) and total small intestine weight (*P* = 0.029). Additionally, fiber and vitamin supplementation affected duodenal and jejunal weights and jejunal length. Lysine restriction alone had minimal impact on intestinal development, but its combination with dietary fiber led to subtle changes in gut morphology. These findings highlight the need for further research on varying lysine levels and their interaction with fiber, particularly in relation to cecal surface area and gut microbiota.

The present findings align with previous studies demonstrating that moderate crude CP reduction does not markedly alter gross intestinal morphology but significantly affects finer histomorphological features. Consistent with Kuritza et al. (2022), Tajudeen et al. (2022), and Song et al. (2023), CP reduction led to significant decreases in epithelial length and villus height. This highlights subtle yet important changes in mucosal architecture. The intestinal lumen diameter, influenced by nutrition, affects the passage and absorption of digested nutrients, while its changes and wall thickening may hinder absorption. Moreover, the observed significant effects of vitamin and insoluble fiber supplementation on epithelial and villus parameters corroborate reports by Moradi et al. (2021), who emphasized the role of dietary fiber in enhancing villus height, gut integrity and nutrient absorption. The significant lysine × fiber and fiber × vitamin interactions observed in this study further suggest that nutrient synergy is critical for maintaining optimal intestinal microstructure under amino acid-restricted conditions. In our study, CP reduction led to a non-significant decrease in the villus height-to-crypt depth (VH:CD) ratio, while villus height and epithelial length were notably reduced (P < 0.05). These findings are consistent with those reported by Tajudeen et al. (2022) and Laudadio et al. (2022), who reported that CP reduction decreased the VH:CD ratio and villus height. But lysine restriction had no significant effect. As villus height is a key indicator of absorptive capacity (Segú et al., 2022), and the VH:CD ratio is positively linked to lactic acid bacteria counts, gut health, and body weight gain, but negatively associated with leg yield (Nguyen et al., 2021), these findings highlight complex nutrient–morphology interactions. The morphometric data were validated by a comparative analysis between conventional (Cell^A) and AI-assisted (ImageJ) methods, which showed strong agreement, ensuring measurement reliability.

Lysine restriction down to 5.7 % of CP did not significantly affect intestinal wall thickness, indicating that CP reduction itself had a stronger direct morphological impact. However, lysine plays multifaceted roles in gut health beyond structure. Deficiency can impair collagen stability (via hydroxylysine synthesis), cellular energy metabolism in the mucosa (via carnitine production), and protein synthesis through mTOR signaling (Mine & Zhang, 2015). It may also compromise immune regulation and antioxidant capacity (Hong et al., 2017). The significant reduction in crypt number under lysine restriction (*P* = 0.002) underscores its specific role in regulating epithelial cell proliferation. Fiber supplementation reduced serosal thickness, likely due to altered nutrient absorption or transit time. Significant Lys × Vit × Fib interactions emphasize the need for balanced nutrient supply to maintain intestinal health. Overall, lysine reduction to 5.7 % appears to be a safe threshold without major adverse effects on intestinal morphology.

Reduced dietary protein significantly decreased crypt length, area, and perimeter, along with goblet cell area, though goblet cell counts remained statistically unchanged—consistent with Kuritza et al. (2022), who reported that crude protein restriction reduced goblet cell numbers (*p* < 0.05). Lysine restriction further exerted a significant depressive effect on both goblet cell counts per crypt and crypt area. Given that goblet cells (∼10 % of gut epithelium) critically maintain intestinal homeostasis via mucin secretion (Kim & Ho, 2010). The observed reduction in goblet cell numbers under lysine restriction can be attributed to the preferential allocation of amino acids toward meeting the nutritional demands of intestinal epithelial cells—such as enterocytes and goblet cells—over other bodily tissues. It is also noteworthy that the synthesis of intestinal mucus is rarely impaired solely due to the deficiency of a single amino acid in the diet (Moran, 2016). The higher goblet cell density observed in the crypts of Lieberkühn compared to the villi (Table 8) may be attributed to their role as the primary site of intestinal stem cell proliferation and differentiation. As the origin of goblet cells, crypts require enhanced mucosal defense, and differentiated cells migrate upwards toward the villus tip. Notably, crude fiber influenced crypt and goblet cell metrics more strongly than vitamins, warranting further study on fiber-specific mechanisms.

Dietary CP reduction significantly decreased enterocyte number, while lysine restriction had no notable effect on enterocyte-related parameters. Vitamin fortification markedly increased both enterocyte count and height, highlighting the critical role of adequate vitamin supply in maintaining intestinal epithelial integrity under CP reduction or amino acid imbalance conditions. Furthermore, the absence of significant interactions indicates independent additive effects of each factor on intestinal morphology, underscoring the importance of simultaneous management of protein and vitamin levels in poultry nutrition. Our findings align with Reboul (2019), showing that vitamin E is absorbed and transported via scavenger receptor class B type I (SR-BI) in enterocytes, which facilitates its movement to the apical membrane and regulates intracellular levels. SR-BI also likely enhances vitamin E absorption indirectly by promoting lipid flux. This absorption mainly occurs in the distal jejunum and ileum. In low-protein or low-amino acid diets, enterocyte proliferation and epithelial thickness in broilers are influenced by protease supplementation (Cardinal et al., 2019) as well as by the type and metabolic fate of amino acids in the gut mucosa. Supplemented (non–protein-bound) amino acids achieve higher and faster concentrations in the portal circulation compared to protein-bound amino acids, thereby exerting a more direct effect on enterocytes (Moss et al., 2018).

Although this study applied conventional two-dimensional morphometry, which remains widely used in poultry gut research, design-based stereology is recognized as the gold standard for unbiased quantitative estimates of volume, surface, and number densities. Future work incorporating stereological sampling would provide more rigorous three-dimensional insights and minimize potential biases of 2D analysis (Mouton, 2002).

## Conclusion and recommendations

This study revealed that while crude CP reduction and lysine restriction exerted limited effects on overall intestinal morphometry (gross morphology), they significantly altered jejunal histoarchitecture, including epithelial length, villus height, and crypt structure. A 12 % CP reduction, particularly with lysine at 5.7 % of CP, exacerbated these changes. Supplementation with insoluble fiber and vitamins notably improved mucosal structure and goblet cell distribution, with significant fiber-vitamin interactions enhancing epithelial integrity. Key strengths include the factorial design and multi-scale histomorphometric analysis. Therefore, reduced-CP broiler diets should be precisely formulated with adequate vitamin fortification and insoluble fiber inclusion to optimize intestinal morphology and support gastrointestinal development and overall performance.

### Study limitations and future perspectives

Although the present morphometric assessment using light microscopy offers a detailed view of jejunal architecture, it is constrained by its reliance on two-dimensional histological sections. A design-based stereological approach would allow for more robust three-dimensional quantification. Moreover, to examine key ultrastructural features such as microvilli density and tight junction organization, higher-resolution imaging techniques are required. The lack of accompanying molecular data—for instance, on the expression of tight junction–related genes—restricts a functional interpretation of the observed epithelial barrier adaptations. Looking ahead, employing advanced electron microscopy (TEM or SEM) could uncover valuable ultrastructural details not accessible via light microscopy. In addition, while this study concentrated on the jejunum, future work might benefit from parallel examination of the cecum—particularly in studies evaluating dietary fiber—using both light and electron microscopy. Ultimately, integrating systematic three-dimensional reconstruction (stereology) with molecular analyses would yield a more holistic understanding of intestinal adaptation mechanisms.

### List of abbreviations

AA: Amino acid

AEZ_T: Apical Epithelial Zone Thickness

BW/SmitL: Body Weight to Small Intestine Length Ratio

BW/SmitW: Body Weight to Small Intestine Weight Ratio

BWG: Body Weight Gain

CD: Crypt Depth

CD/CP: Crypt depth to crude protein intake ratio

CD/ST: and crypt depth to starch intake ratio

CecL: Cecum Length

CecW: Cecum Weight

CrArea: Crypt Area

CrGbAr: Crypt Goblet Cell Area

CrGbN: Crypt Goblet Cell Number

CR_BsWd: Crypt Basal Width

CR_Len: Crypt Length

CR_Prm: Crypt Perimeter

CryptN: Number of Crypts

CSA: Cecal Surface Area

CP: Crude Protein

DCAB: Dietary cation–anion balance

DeL: Duodenum Length

Dew: Duodenum Weight

ENS: Enteric nervous system

Ent_Cnt: Number of Enterocytes in Field

Ent_Dns: Enterocyte Density

Ent_HA: Average Enterocyte Height on Villus

EpiLen: Epithelium Length

FCR: Feed Conversion Ratio

FI: Feed Intake

Fib: Insoluble Fiber

GbCrRt: Goblet to Crypt Ratio

GbVLH: Goblet Cells per Villus Height

GbVLRt: Goblet Area to Villus Head Area Ratio

Gob_Cnt: Number of Goblet Cells in Field

Gob_CR: Goblet Cell Number per Crypt

Gob_Dens: Goblet Cell Density

GobHt: Average Goblet Cell Height

H: High Lysine /CP Ratio (6.2 %)

HV1F1: High Lysine + Standard Vitamin + Fiber

HV2F0: High Lysine + Extra Vitamin, No Fiber

ILW: Inner Lumen Width

ILD: Inner Lumen Diameter

ILA: Inner Lumen Area

ILP: Inner Lumen Perimeter

ILeW: Ileum Weight

IleL: Ileum Length

JejL: Jejunum Length

JejW: Jejunum Weight

L: Low Lysine/CP Ratio (5.7 %)

LuCrptNo: Luminal Crypt Number

Lys: Lysine

MuslTh: Muscular Layer Thickness

OLW: Outer Lumen Width

OLD: Outer Lumen Diameter

PAS: Periodic Acid-Schiff

RCP: Reduced crude protein

SCFA: Short-chain fatty acid

SeroTh: Serosa Thickness

SEM: Standard Error of the Mean

SGLT1: Sodium-dependent glucose transporter 1

SmitL: Small Intestine Length

SmitW: Small Intestine Weight

SubmuTh: Submucosa Thickness

TotalTh: Total Thickness (Serosa + Muscle + Submucosa)

VH: Villus Height

VH/CD: Villus Height to Crypt Depth Ratio

VH/CP: Villus height to crude protein intake ratio

VH/ST: Villus height to starch intake ratio

Vit: Vitamin

VN: Villus Number

VSA: Villus Surface Area

VW: Villus Width

VWm: Villus Width (Middle)

VWtp: Villus Width (Top)

V1: Standard Vitamin Supplementation

V2: Extra Vitamin Supplementation

ZO-2 gene: Zonula occludens-2 gene

## Funding

This research received no external funding.

## Institutional review board statement

Not applicable.

## Data availability statement

Not applicable.

## CRediT authorship contribution statement

**Ahmad Salahi:** Writing – review & editing, Writing – original draft, Visualization, Validation, Software, Methodology, Investigation, Funding acquisition, Formal analysis, Data curation, Conceptualization. **M.H. Shahir:** Writing – review & editing, Validation, Supervision, Project administration, Methodology, Investigation, Formal analysis, Conceptualization. **Iraj Jafari Anarkooli:** Writing – review & editing, Validation, Supervision. **Zahra Abdi:** Writing – review & editing, Supervision, Project administration, Methodology.

## Declaration of competing interest

The authors declare no competing interests.

## Appendix Supplementary materials

Image, application 1
